# COVID-19 and Its Potential Impact on Achieving Pediatric Hospital Medicine Board Certification for Established Internal Medicine-Pediatrics (Med-Peds) Hospitalists

**DOI:** 10.7759/cureus.54708

**Published:** 2024-02-22

**Authors:** Andrea M Lauffer, Angela Noble, David Fish, Rachel Peterson, Madeleine Matthiesen

**Affiliations:** 1 Hospital Medicine, Marshall University Joan C. Edwards School of Medicine, Huntington, USA; 2 Hospital Medicine, Allina Health, Minneapolis, USA; 3 Internal Medicine-Pediatrics, University of Massachusetts, Worcester, USA; 4 Hospital Medicine, Cincinnati Children’s Hospital Medical Center, Cincinnati, USA; 5 Hospital Medicine, Massachusetts General Hospital, Boston, USA

**Keywords:** med-peds, change in practice, covid-19, board-certified, pediatric hospital medicine

## Abstract

With the onset of the COVID-19 pandemic and the subsequent rise in adult hospitalized patients, many internal medicine and pediatrics (Med-Peds) hospitalists volunteered or were required by their hospital employers to transition their practice to caring for hospitalized adult patients only. Some Med-Peds hospitalists had a disruption in their practice that may affect their board eligibility for the pediatric hospital medicine (PHM) subspecialty exam in 2024. In this editorial, we review the rise of Med-Peds hospitalist careers, the evolution of PHM becoming a subspecialty, and the effect of the COVID-19 pandemic on some Med-Peds hospitalists in their quest to become board certified in PHM via the practice pathway.

## Editorial

With the onset of the COVID-19 pandemic and the subsequent rise in adult hospitalized patients, many internal medicine-pediatrics (Med-Peds) hospitalists volunteered or were required by their hospital employers to transition their practice to caring for hospitalized adult patients only. With the drop in the inpatient pediatric census, some institutions even recruited pediatric hospitalists to help care for the volume of adult hospitalized patients [1].

Currently, the practice pathway is the only path in which established Med-Peds hospitalists can obtain pediatric hospital medicine (PHM) board eligibility. With hospital medicine becoming an increasingly popular field of practice among Med-Peds-trained physicians over the past several years, those with experience may lose the opportunity to become board certified in PHM due to shifts in practice experienced during the COVID-19 pandemic and the closure of the practice pathway in 2024. The only path to becoming board eligible after 2024 will be returning as a trainee by matching into and completing a dedicated PHM fellowship program. It is uncertain whether facilities may shift to hiring exclusively board-eligible or board-certified PHM hospitalists. As a result of this uncertainty, it can be categorized as a threat for some Med-Peds hospitalists who still want to practice in the field of PHM but may be limited in opportunities due to possible future standards requiring them to be either PHM board eligible or PHM board certified. This may limit Med-Peds hospitalists' ability to practice PHM and reduce hiring opportunities for Med-Peds hospitalist candidates in academic and community-based hospitals. The consequence of this has the potential to harm the hospitalist work field among Med-Peds-trained physicians.

Med-Peds physicians play a relevant role in the field of hospital medicine. Their role in navigating their practice in the field of hospital medicine has become even more highlighted with published studies of Med-Peds workforce characteristics, the birth of the PHM subspecialty board certification, and their utility as both internal medicine and pediatric hospitalists during the COVID-19 pandemic. 

In 2015, the American Academy of Pediatrics (AAP) released the results of a Med-Peds workforce survey. About 22.4% of Med-Peds graduates were identified as practicing hospitalists [2]. A study published in 2022 reviewed the workplace characteristics of Med-Peds hospitalists. From the survey, 40 hospitalists from 24 institutions saw both adult and pediatric patients the same day [3]. This study also highlighted that 34.9% of the institutions surveyed were required to provide adult-based care within the pediatric hospital due to the COVID-19 pandemic [3].

In 2013, leaders from the PHM National Leaders Conference convened to determine if petitioning for a PHM subspecialty was needed. It was at this conference that the decision was made to create a two-year subspecialty certification [4]. In 2014, a group from the Joint Council on Pediatric Hospital Medicine petitioned the American Board of Pediatrics (ABP) for a subspecialty certification [5]. When presented to the ABP, an 18-month review took place. After it was approved by the ABP Board of Directors and then by the American Board of Medical Specialties, the ABP appointed a PHM sub-board who went on to establish the eligibility criteria via three pathways (practice, training, and combination). The Accreditation Council for Graduate Medical Education then approved the PHM training program requirements. Finally, the PHM sub-board wrote questions for the initial certifying exam [6].

PHM became the 15th pediatric subspecialty offered by the ABP [6]. The first exam was offered in 2019. A total of 1,500 pediatric hospitalists took the exam [4]. Of the 1,627 applicants, 93% were approved to sit for the exam [6]. The training pathway required completion of a two-year PHM fellowship. The combined pathway offered eligibility to those who had completed less than two years of fellowship training but had an additional two years of clinical experience. The practice pathway was open to those who had four years of clinical experience in PHM [7]. The pass rate for the initial PHM exam in 2019 was 84.2% [6].

For Med-Peds hospitalists with years of experience, the practice pathway become their avenue for achieving board certification. Starting with the graduating residency classes of 2020, the practice pathway eligibility was closed. To qualify for the 2019 initial exam via the practice pathway, hospitalists had to have four years of clinical experience by June 30, 2019, and they had to meet a certain number of clinical hours caring for hospitalized pediatric patients [8]. With the advent of the COVID-19 pandemic, the ABP altered the requirements for hospitalists who were seeking board eligibility through the practice pathway. A major alteration in the practice pathway was extending the look back window from four to five years of clinical practice due to the COVID-19 pandemic [9]. A total of 900 hours per year of practice dedicated to PHM is required for board eligibility. Med-Peds hospitalists are now required to spend greater than or equal to 450 hours per year every year for four out of the five years in the direct clinical care of hospitalized children. The additional requirement of 450 work hours can be counted toward the direct care of hospitalized children, but not toward the care of hospitalized adults. Additional hours can be devoted to administration, research, or teaching activities related to the care of hospitalized children. Activities in quality improvement and patient safety can also be counted and be related to the care of hospitalized children and/or adults [10].

To assess the impact of these changes, Med-Peds representatives from the Society of Hospital Medicine and the AAP created a survey that was distributed nationally to Med-Peds hospitalists between February 2023 and June 2023. Of note, the survey was not sponsored by either the AAP or the Society of Hospital Medicine. Institutional Review Board approval was obtained to create and distribute the survey. The survey was administered via RedCap and distributed on the AAP Med-Peds listsserv, advertised via Twitter (now X), and given to Med-Peds special interest group committee leaders. Participants remained anonymous. In total, 39 surveys were completed.

Respondents’ clinical time dedicated to PHM was assessed prior to the COVID-19 pandemic. An assessment of the decrease in PHM time was also analyzed for the 11 respondents who experienced such a practice shift. Of the 11 respondents, eight stated that they both volunteered and were under mandatory obligation to decrease their PHM time. One respondent’s decrease was voluntary. Two were mandatory. The total PHM disruption time for the two respondents' mandatory shift was >24 months and 10-12 months. The total PHM disruption time for both voluntary and mandatory was composed of the following: four respondents = 7-9 months, two respondents = 21-24 months, one respondent = 10-12 months, and one respondent = 19-21 months. The total PHM disruption time for the voluntary respondent was 10-12 months. For 25 respondents, PHM practice remained the same pre- and post-COVID-19 pandemic. Eight respondents stated that their PHM practice is now increased compared to pre-COVID-19 pandemic. Six respondents stated that their PHM time is now decreased compared pre-COVID-19 pandemic.

Participants of the survey were asked if practice changes related to the pandemic could affect their board eligibility for the PHM exam in 2024. Of the 39 participants, five said yes. In an attempt for a qualitative assessment, participants were allowed to leave open-ended comments with regard to how the creation of PHM board exam has affected their practice and career pursuits in light of the COVID-19 pandemic. Table 1 highlights some of those comments. 

**Table 1:** Survey participants' open-ended comments with regard to how the creation of the PHM board exam affected their practice and career pursuits in light of the COVID-19 pandemic Med-Peds: Internal medicine and pediatrics; PHM: pediatric hospital medicine

Respondent comments on the PHM board qualification due to the COVID-19 pandemic
“Was considering PHM boards (and still am) but the pandemic set me back and would appreciate more time to decide, register, and take the test.”
“Only reason I could keep 25% of my hospitalist time in Peds during COVID was intentional support from leadership to maintain this minimum for Med-Peds hospitalists considering PHM certification."
“Plan to sit for 2024 exam, hopeful that ABP finds me qualifying, but concerned that being deployed to IM for three months (Mar-Jun 2020) will hinder that.”
“…Med-Peds docs helped children's hospitals with protocols to care for young adults when the children's hospitals offered to help care for older patients. During the time when pediatric volumes were quite low, some pediatric-only/children's hospital assignments were changed to adult assignments to meet the needs of both hospitals. It would not be surprising if this were a larger theme nationwide."

The unforeseen event of the COVID-19 pandemic has created a barrier for some practicing Med-Peds hospitalists in the country to achieve PHM board certification via the practice pathway. While the majority of our survey participants did not experience a negative impact in their PHM career journey, there were ultimately some Med-Peds physicians who did.

The evolvement of the field of PHM on existing Med-Peds hospitalists is not well studied which creates limitations in assessing long-term career impacts for those who want to pursue PHM board certification. Furthermore, the compounding factor of the COVID-19 pandemic soon after the inaugural 2019 PHM board exam was administered has further complicated the ability to assess barriers for PHM board eligibility for Med-Peds hospitalists. While our study sample was small for our survey and is a noted limitation, it did reveal that the COVID-19 pandemic has presented additional challenges for some Med-Peds hospitalists to pursue PHM board eligibility.

The practice pathway eligibility will end in 2024. We hope that the ABP will take into consideration the sect of Med-Peds hospitalists that were deployed to care for adults during this historic national health crisis and allow an adequate opportunity for them to achieve board eligibility before permanent closure of the practice pathway.

## References

[1] Osborn R, Doolittle B, Loyal J (2021). When pediatric hospitalists took care of adults during the COVID-19 pandemic. Hosp Pediatr.

[2] Donnelly MJ, Lubrano L, Radabaugh CL, Lukela MP, Friedland AR, Ruch-Ross HS (2015). The Med-Peds hospitalist workforce: results from the American Academy of Pediatrics Workforce Survey. Hosp Pediatr.

[3] Moza R, Fish D, Peterson RJ (2022). Workforce characteristics of Med-Peds hospitalists. Cureus.

[4] Roberts KB, Fisher ER, Rauch DA (2020). The history of pediatric hospital medicine in the United States, 1996-2019. J Hosp Med.

[5] Barrett DJ, McGuinness GA, Cunha CA (2017). Pediatric hospital medicine: a proposed new subspecialty. Pediatrics.

[6] The American Board of Pediatrics (2019). ABP 2019 annual report. ABP 2019 annual report.

[7] Rosh Review (2019). Everything You Need To Know for the Pediatric Hospital Medicine Certification Exam. https://www.roshreview.com/blog/everything-you-need-to-know-for-the-pediatric-hospital-medicine-certification-exam/.

[8] Otto Otto, MA MA (2019). Pediatric hospitalist certification beset by gender bias concerns. The Hospitalist.

[9] (2023). Frequently Asked Questions When Applying for Subspecialty Certification in Pediatric Hospital Medicine (PHM). https://www.abp.org/sites/abp/files/pdf/phm-faqs.pdf.

[10] (2023). Pediatric Hospital Medicine Certification. https://www.abp.org/content/pediatric-hospital-medicine-certification.

